# The Prognostic Impact of Imaging Detected Tumor Deposits in Rectal Cancer: A Systematic Review and Meta-analysis

**DOI:** 10.1245/s10434-025-18371-w

**Published:** 2025-09-30

**Authors:** Harpreet Kaur Sekhon Inderjit Singh, Amy Lord, Nikhil Pawa, Gina Brown

**Affiliations:** 1 Department of Surgery and Cancer, Imperial College London, London, UK; 2 Department of Surgery, Buckinghamshire NHS Trust, Oxford, UK; 3 Department of Surgery, Basingstoke and North Hampshire Hospital, Basingstoke, UK; 4 Department of Surgery, Chelsea and Westminster NHS Trust, London, UK

**Keywords:** Rectal cancer, Tumor deposit, Magnetic resonance imaging, Prognostic factor

## Abstract

**Background:**

Tumor deposits (TDs) identified at pathology post rectal cancer resection are increasingly recognized as an adverse prognostic factor. Advances in magnetic resonance imaging (MRI) allow for the preoperative detection of TDs (mrTDs), raising the possibility that patients with mrTDs may benefit from neoadjuvant treatment strategies or enhanced surveillance.

**Objective:**

This meta-analysis primarily aims to evaluate the prognostic significance of mrTDs in rectal cancer patients. We also evaluate the impact of other prognostic features.

**Methods:**

A comprehensive search was conducted in MEDLINE, Embase, and Scopus databases. The Preferred Reporting Items for Systematic Reviews and Meta-Analyses (PRISMA) guidelines were followed. Prognostic endpoints included disease-free survival (DFS) and overall survival (OS). Pooled hazard ratios (HR) were calculated using R software.

**Results:**

Overall, 5813 and 5630 patients were included in the systematic review (13 studies) and meta-analysis (11 studies), respectively. The pooled HR for OS and DFS for mrTDs was 2.1 (95% confidence interval [CI] 1.63–2.70; *p* < 0.0001) and 2.13 (95% CI 1.68–2.71; *p* < 0.0001), respectively. The pooled HR for OS and DFS for extramural venous invasion (mrEMVI) and circumferential resection margin (mrCRM) involvement was also significantly increased (*p* < 0.01). The pooled HR for MRI-predicted nodal disease (mrLNMs) and T stage (mrT) for OS and DFS was insignificant (*p* > 0.05).

**Conclusion:**

This meta-analysis confirms the prognostic significance of mrTDs on OS and DFS in rectal cancer. This was also observed for mrEMVI and mrCRM, but not for mrLNMs or mrT. Current staging using TNM has poor prognostic power. A novel evidence-based staging system is needed to identify high risk patients and guide preoperative treatment strategies.

*Trial registration* The protocol for this study was registered in the PROSPERO database (CRD420251011285)

**Supplementary Information:**

The online version contains supplementary material available at 10.1245/s10434-025-18371-w.

Preoperative identification of rectal cancer patients at high risk of recurrence or death remains an unmet clinical challenge. Beyond the traditional tumor (T), node (N), and metastasis (M) stages, other factors are increasingly being recognized as important in predicting prognosis. One such factor is tumor deposits (TDs), which are defined by the Union for International Cancer Control (UICC) as deposits of tumor cells separate to the main body of the tumor within the peri-rectal fat without evidence of any lymphatic, vascular, or neural tissue.^1^ When identified on histopathology (pTDs), pTDs are present in approximately 22% of colorectal cancer specimens^2,3^ and are associated with significantly poorer oncological outcomes, including a near doubling of the odds of developing liver and peritoneal metastases in node-positive patients.^3–5^

In modern management of rectal cancer, treatment strategy is predominantly determined prior to surgery and as such, radiological staging using magnetic resonance imaging (MRI) forms the cornerstone of decision making.

Standard MRI staging has historically emulated the pathological TNM system, but whether this maintains equivalent prognostic power is controversial. MRI has been shown to accurately predict a safe circumferential resection margin (CRM)^6^ and recognize extramural venous invasion (EMVI), which are two factors increasingly recognized as strong predictors of poor prognosis.^7^ In contrast, MRI appears to be less accurate at detecting lymph node metastases (LNMs).^8,9^ More recently, MRI detection of TDs (mrTDs) has also been described as a potential imaging biomarker. In 2022, Lord et al. were the first to describe mrTDs as irregular nodules within the mesorectum that appear to directly interrupt the course of veins, are discontinuous from the primary tumor, and are radiologically distinct from suspected LNMs.^10^ Since then, several retrospective studies have suggested that mrTDs reflect a similar poor prognostic outcome as pTDs and are superior to T and N stages in predicting prognosis.^10,11^ The three-dimensional nature of imaging over two-dimensional histopathology has also been hypothesized to confer an advantage in TD detection.

To date, no meta-analysis has evaluated the prognostic impact of mrTDs in rectal cancer. This systematic review and meta-analysis therefore primarily aims to achieve this. As well as assessing the association between mrTDs and prognosis, we aim to put this into context by comparing this with the prognostic impact of other reported MRI staging parameters. We hope that this work adds to the increasing body of evidence to support the routine radiological reporting of mrTDs and their inclusion in a more evidence-based staging system that guides risk stratification and neoadjuvant and surveillance management decisions in these patients.

## METHODS

### Search Strategy

This systematic review and meta-analysis was performed in accordance with the Preferred Reporting Items for Systematic Reviews and Meta-Analyses (PRISMA) guidelines.^12^ A comprehensive literature search was performed using a combination of free-text and Medical Subject Heading (MeSH) terms within the MEDLINE, Embase, and Scopus databases. The detailed search strategy is provided in electronic supplementary material (ESM) Table S1. All abstracts, studies, and reference lists identified were reviewed. No restrictions were made on publication year or article type. Only studies published in English were included in the search strategy. The latest date for this search was 10 March 2025.

### Eligibility Criteria

All published studies were eligible for inclusion if they reported on:

1. adults with rectal cancer;
2. mrTDs;
3. prognosis. Our primary measures of prognosis included disease-free survival (DFS) and overall survival (OS), while our secondary measures of prognosis included local recurrence (LR) and distant metastases (DM) as these are less commonly reported oncological outcomes. As a result, we do not expect LR and DM to be included in the meta-analysis but rather to discuss them in the review.

DFS, OS, LR, and DM are defined as the time from surgery to cancer recurrence (local or distant); time from surgery to death from any cause; the development of cancer recurrence locally post-surgery; and the development of cancer spread to distant organs/sites after surgery, respectively. In this manuscript, the prefix ‘mr’ will be used to denote the identification of T stage, LNMs, TDs, EMVI, and CRM on MRI.

### Data Extraction and Statistical Analysis

Two review authors (HKSIS and NP) independently examined and determined the eligibility of all studies. The following data were extracted from the included studies: first author’s name, year of publication, inclusion and exclusion criteria, study design, sample size, mrTD definition used, participant characteristics (age, sex, type and stage of cancer), duration of follow-up, presence or absence of mrTDs, mrLNMs, mrEMVI and mrCRM involvement and prognosis. Discrepancies between reviewers were resolved through discussion and further review by a third reviewer (AL). The risk of bias of the included studies was assessed using the Newcastle–Ottawa Scale (NOS). Where hazard ratios (HRs) and 95% confidence intervals (CIs) on multivariate analyses were not available, respective authors were contacted via email in an attempt to obtain this information. Univariate data were not utilized to ensure control for confounding variables and improve methodological rigor.

A random effects meta-analysis was conducted using R software v4.3.2 and the ‘meta’ package. A random-effects model was chosen to account for between-study variability, even in cases of low heterogeneity, as clinical and methodological diversity was anticipated. Publication bias was assessed visually using funnel plots. Heterogeneity within studies was assessed with forest plots, using the Chi-square test, and quantified by means of the inconsistency index (I^2^). I^2^ values of 25%, 50%, and 75% were considered low, moderate, and high heterogeneity, respectively.

## RESULTS

### Study Selection and Baseline Characteristics

**Fig 1:**
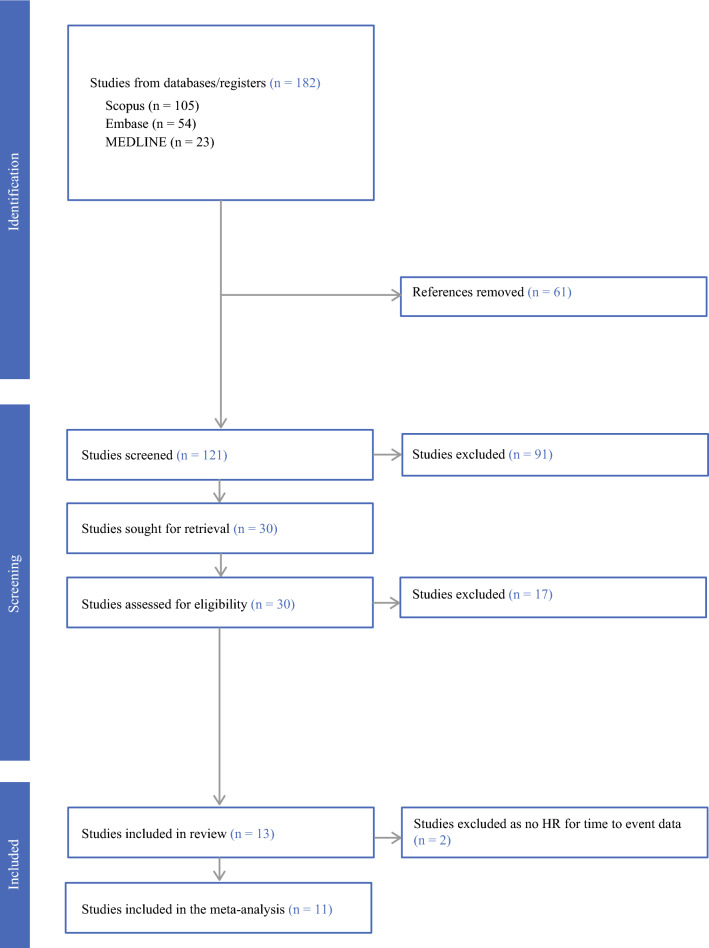
PRISMA flowchart. *PRISMA* Preferred Reporting Items for Systematic Reviews and Meta-Analyses

A total of 182 references were identified. Duplicates and obviously irrelevant abstracts were excluded at title and abstract level, leaving 30 articles that were reviewed fully. Seventeen articles were excluded after full review because of wrong outcome, intervention, or patient population. The remaining 13 studies were included in the systematic review (Fig. 1).^10,11,13–23^ Baseline characteristics of the included studies with risk-of-bias assessment are provided in Table 1. All studies were retrospective in nature and investigated rectal cancer patients. Two studies from the review could not be included in the meta-analysis—one because *p*-values only were provided without the HR for time-to-event outcomes,^23^ and the other because it was the only study that utilized odds as a measure of association.^22^ Of the 11 studies included in the meta-analysis, 10 looked at DFS, 9 at OS, 1 at LR, and 2 at DM (Table 1). As expected, the results for LR and DM were therefore only discussed as part of the systematic review.

**Table 1:** Baseline characteristics of the included studies

First author	Year of publication	Country	Design	Inclusion period	Rectal versus colon	Cancer stage	Total no. of cases	Neoadjuvant treatment (%)	mrTD definition provided	Multivariate mrTD prognostic data available	Median follow-up, in months (IQR)	NOS
Lord ^10^	2022	UK	Retrospective, multicenter	2007–2015	100% rectal	1–3	233	52	Yes	DFS, OS	61 (0–132)	8
Di Fabio^11^	2024	UK	Retrospective, single center	2008–2020	100% rectal	1–3	314	17	Yes	DFS, OS^b^	64	8
Marjasuo^13^	2024	Finland	Retrospective, single center	2017–2018	100% rectal	1–3	480	57.7	Yes	DFS, OS	42 (33–56)	9
Geffen^14^	2024	Netherlands	Retrospective, cross-sectional population-based	2016	100% rectal	2–3	1214	81.6	Yes	DFS, OS, LR, DM	48 (30–54)	8
Chandramohan^15^	2022	India	Retrospective, single center	2012–2018	100% rectal	2–3	297	100	Yes	DFS, OS	49.3 (6.6–101)^c^	7
Zhao^16^	2023	China	Retrospective, single center	2011–2017	100% rectal	2–3	328	100	Yes	OS	79 (66–94)	7
Schaap^17^	2021	Netherlands	Retrospective, single center	2009–2015	100% rectal	2–3	277	100	Yes	DFS, DM	47.6 (34.2–62.8)	8
Que^18^	2024	China	Retrospective, single center	2014–2020	100% rectal	2–3	91	100	Yes	DFS^b^	31 ( 2–88)	7
Hu^19^	2025	China	Retrospective, single center	2013–2018	100% rectal	2–3	947	57.2	Yes	DFS, OS	56 (45.0–72.3)	8
Huang^20^	2025	China	Retrospective, multicenter	2009–2018 (varied depending on the center)	100% rectal	2–3	1188	100	Yes	DFS, OS	37.8 (19.6–51.8)	8
Lord^21^	2022	UK	Retrospective, multicenter	2007–2017 (varied depending on the center)	100% rectal	1–3	378	0	Yes	DFS, OS	66 (44–95)	8
Lv^22 a^	2023	China	Retrospective, single center	2014–2019	100% rectal	2–3	114	0	Yes	DM	24	8
Mayaud^23 a^	2022	France	Retrospective, single center	2010–2014	100% rectal	2–3	69	100	Yes	DFS, OS	NS	7

*mrTD* MRI-detected tumor deposit, *MRI* magnetic resonance imaging, *IQR* interquartile range, *DFS* disease-free survival, *OS* overall survival, *LR* local recurrence, *DM* distant metastases, *NOS* Newcastle–Ottawa Score, *NS* not stated

^a^ Included in the systematic review only

^b^ Data provided by the authors on request

^c^ Mean follow-up

A total of 5813 and 5630 patients were included in the systematic review and meta-analysis, respectively; 64.6% of all patients were male. The mean prevalence of mrTD, mrEMVI, mrLNM, and mrCRM positivity was 19.9%, 32.8%, 64.1%, and 28.3% respectively (Table 2). Ten of the 11 studies included in the meta-analysis either directly referenced or defined mrTDs verbatim as discontinuous from the main body of the tumor and interrupting the course of a vein, as originally described by Lord and colleagues.^10^ The remaining study described mrTDs as ‘irregular deposits often related to or located within a vessel’.^14^ While not using Lord’s exact wording, this definition is closely aligned conceptually and can reasonably be interpreted as describing the same phenomenon. Five of 11 studies published their MRI protocols, which stated the use of high-resolution diffusion weighting, which is required for optimal identification of mrTDs.^16,18–21^

**Table 2:** Prevalence of MRI-detected high-risk factors in the included studies

First author	Male (%)	Female (%)	mrTDs (%)	mrEMVI (%)	mrCRM (%)	mrLNM (%)
Lord^10^	144 (61.8)	89 (38.2)	84 (36)	119 (51)	76 (33)	98 (42)
Di Fabio^11^	200 (63.7)	114 (36.3)	53 (16.9)	97 (30.9)	38 (12.1)	133 (42.4)
Marjasuo^13^	263 (54.8)	217 (45.2)	81 (16.9)	112 (23.3)	NS	241 (50.2)
Geffen^14^	804 (66.2)	410 (33.8)	179 (14.7)	324 (36.7)	NS	856 (70.5)
Chandramohan^15^	186 (62.6)	111 (37.4)	141 (47.5)	147 (49.5)	NS	181 (61.1)
Zhao^16^	232 (70.7)	96 (29.3)	31 (9.5)	96 (29.3)	82 (25.0)	220 (67.1)
Schaap^17^	166 (59.9)	111 (40.1)	103 (37.2)	163 (58.8)	216 (78.0)	209 (75.5)
Que^18^	66 (72.5)	25 (27.5)	37 (40.7)	59 (64.8)	73 (80.2)	74 (81.3)
Hu^19^	627 (66.2)	320 (33.8)	130 (13.7)	346 (36.5)	132 (13.9)	746 (78.8)
Huang^20^	792 (66.7)	396 (33.3)	216 (18.2)	330 (27.8)	423 (35.6)	835 (70.3)
Lord^21^	231 (61)	147 (39)	78 (21)	90 (24)	24 (6)	126 (33)
Lv^22 a^	76 (66.7)	38(33.3)	30 (26.3)	35 (30.7)	16(14.0)	36 (31.6)
Mayaud^23 a^	46 (66.7)	23 (33.3)	15 (21.7)	27 (39.1)	35 (50.7)	47 (68.1)
Percentage proportion/prevalence (range)	**64.6 (54.8–72.5)**	**35.4 (27.5–45.2)**	**19.9 (9.5-47.5)**	**32.8 (23.3-64.8)**	**28.3 (6.0-80.2)**	**64.1 (31.6-81.3)**

The Bold proportion and range of all the data in the column

*mrTDs* MRI-detected tumor deposits, *mrEMVI* MRI-detected extramural venous invasion, *mrCRM* MRI-detected circumferential resection margin involvement, *mrLNM* MRI-detected lymph node metastases, *MRI* magnetic resonance imaging, *NS* not stated

^a^Included in the systematic review only

According to the NOS for cohort studies, all 13 included studies were of good quality (Table 1). Funnel plots did not show any significant evidence of publication bias (ESM Fig. S1).

### Survival Outcomes: Overall Survival and Disease-Free Survival

**Fig 2:**
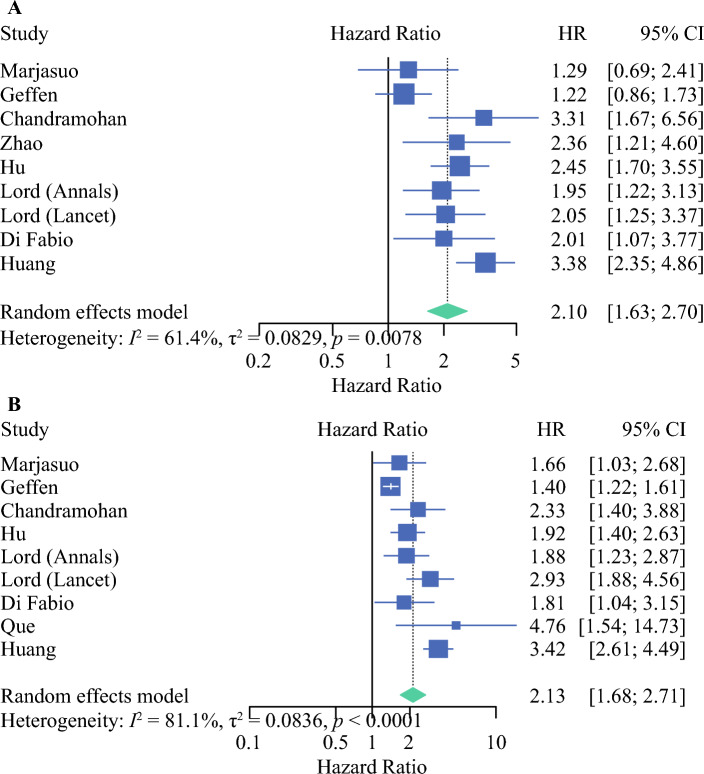
Forest plot showing the effect of mrTDs on (**A**) overall survival (*p* < 0.0001) and (**B**) disease-free survival (*p* < 0.0001). *mrTDs* magnetic resonance imaging-detected tumor deposits, *HR* hazard ratio, *CI* confidence interval

Nine studies reported on the hazard of mrTDs on OS and DFS. Our meta-analysis of these studies confirmed a significantly worse prognosis (Fig. 2). The pooled HR for adverse OS was 2.1 (95% CI 1.63–2.70; *p* < 0.0001). The pooled HR for adverse DFS was 2.13 (95% CI 1.68–2.71; *p* < 0.0001). In 2021, Schaap et al. grouped mrTDs with mrEMVI and evaluated their impact on DFS. On multivariate analysis, they reported a significantly increased risk (*p* < 0.05) when compared with patients without mrEMVI. As the comparators are different, this was not included in the meta-analysis. The study by Mayaud et al., which was not included in the meta-analysis as HRs were not provided, also suggested a significantly adverse DFS (*p* = 0.03) at 3 and 5 years.^23^

**Fig 3:**
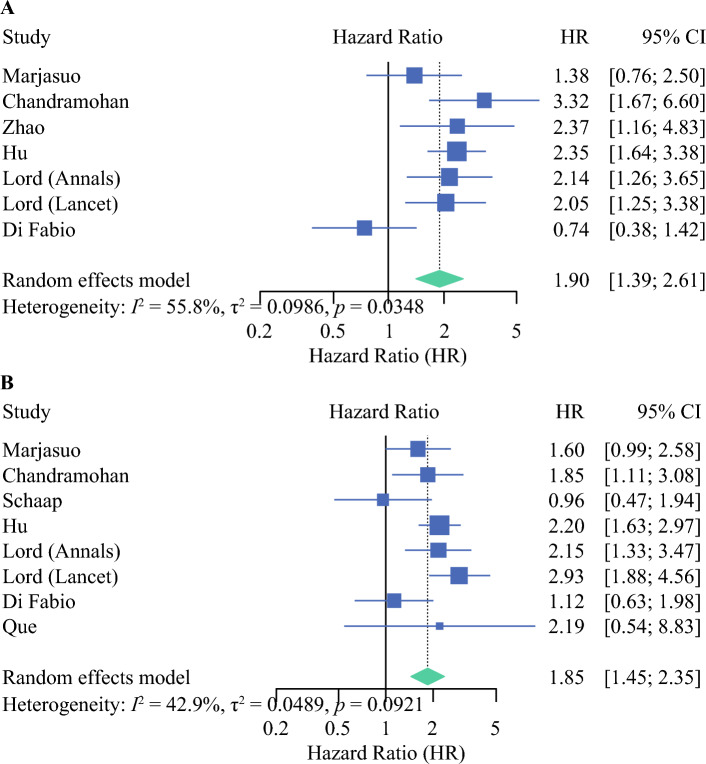
Forest plot showing the effect of mrEMVI on (**A**) overall survival (*p* < 0.0001) and (**B**) disease-free survival (*p* < 0.0001). *mrEMVI* magnetic resonance imaging-detected extramural venous invasion, *HR* hazard ratio, *CI* confidence interval

Seven studies reported on the hazard of mrEMVI on OS, and eight studies reported on its impact on DFS. Our meta-analysis of these studies confirmed a significantly worse prognosis (Fig. 3). The pooled HR for adverse OS and DFS was 1.90 (95% CI 1.39–2.61; *p* < 0.0001) and 1.85 (95% CI 1.45–2.35; *p* < 0.0001), respectively.

Nine studies reported on the impact of T stage on DFS and OS; however, due to variations in the stage of cancer included in the 11 studies and author choice of comparator groups, e.g. mrT2 vs. mrT3 or mrT3-4 vs. mrT2, only three studies were comparable (mrT2-3 vs. mrT3-4). The pooled HRs for OS and DFS were insignificant (ESM Figs. 2 and 3). The studies by Zhao et al. and Geffen et al. were the only two that suggested a significant difference in prognosis when comparing mrT3b-T4 with mrT2-T3a (OS), and mrT4 or mrT3CRM threatened with mrT3CRM not threatened (DFS and OS), respectively.^14,16^

**Fig 4:**
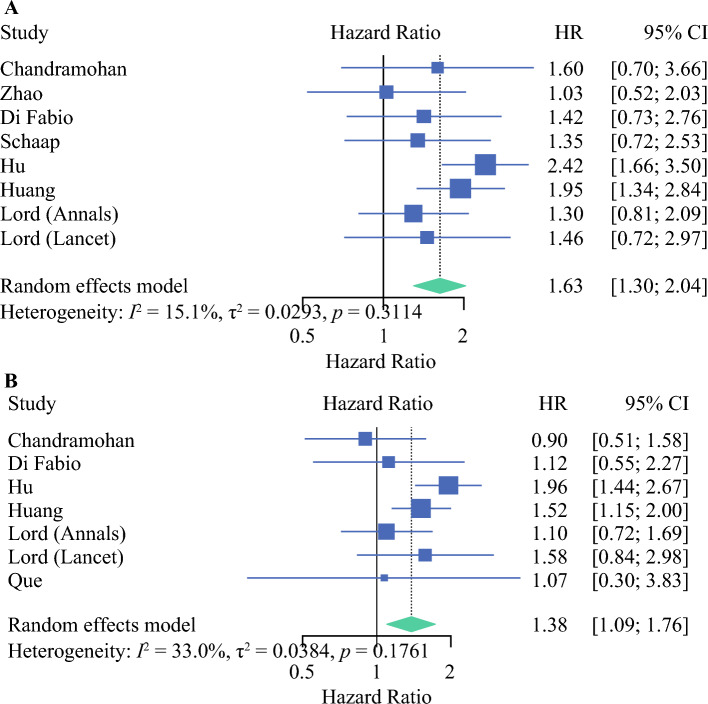
Forest plot showing the effect of mrCRM on (**A**) overall survival (*p* < 0.0001) and (**B**) disease-free survival (*p* = 0.007). *mrCRM* magnetic resonance imaging-detected circumferential resection margin, *HR* hazard ratio, *CI* confidence interval

Eight and 7 studies reported on the hazard of mrCRM involvement on OS and DFS, respectively. Our meta-analysis of these studies confirmed a significantly worse prognosis (Fig. 4). The pooled HRs for adverse OS and DFS were 1.63 (95% CI 1.3–2.04; *p* < 0.0001) and 1.38 (95% CI 1.09–1.76; *p* = 0.007), respectively.

**Fig 5:**
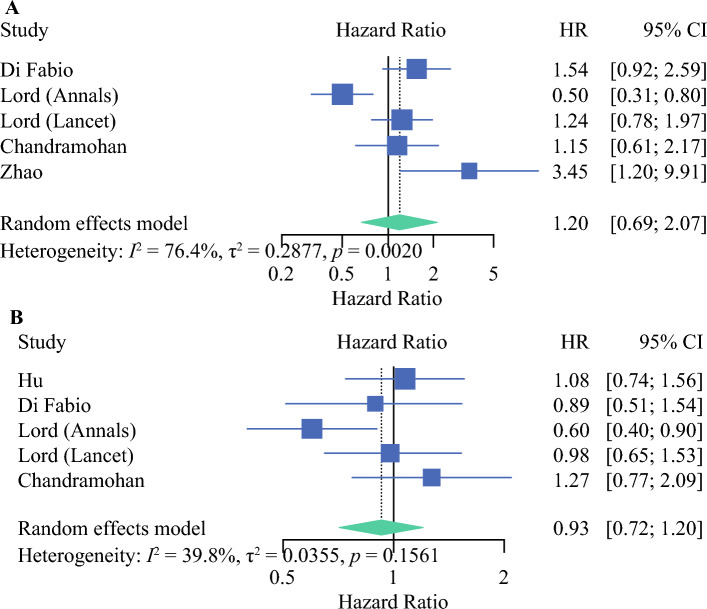
Forest plot showing the effect of mrLNMs on (**A**) overall survival (*p* = 0.40) and (**B**) disease-free survival (*p* = 0.56). *mrLNMs* magnetic resonace imaging-detected lymph node metastases, *HR* hazard ratio, *CI* confidence interval

Five studies reported on the hazard of mrLNM positivity (yes vs. no) on OS and DFS. Our meta-analysis of these studies showed no significant difference in prognosis (Fig. 5). The pooled HRs for adverse OS and DFS were 1.20 (95% CI 0.69–2.07; *p* = 0.40) and 0.93 (95% CI 0.72–1.20; *p* = 0.56), respectively. Three studies compared mrN2 versus mrN1 versus mrN0 in relation to DFS.^14,17,18^ All three showed no significant difference in DFS.

### Patterns of Recurrence: Local Recurrence and Distant Metastases

Only one study reported multivariable HRs for LR and DM outcomes for mrTDs.^14^ Geffen et al. suggested an 81% significantly increased risk of LR and 59% increased risk of DM. Lv et al., who utilized odds ratios as their outcome measure, suggested a 10.15 (*p* < 0.05) increased risk of developing DM if mrTDs were present.^22^ Only one study evaluated the impact of mrEMVI on DM.^17^ This suggested a 43% significantly increased risk of DM. Zhao et al. analyzed T-stage impact on DM, suggesting a 100% increased risk (*p* = 0.009) of DM in mrT3b-T4 patients compared with mrT2-T3a patients.^16^ Geffen et al. evaluated the impact of mrT4 and mrT3CRM not threatened disease with mrT3CRM threatened disease on LR and DM. There was no significant difference for LR (OR mrT3CRM not threatened 1.29; OR T4 1.75; *p* = 0.161). There was a 26% and 58% increased risk of DM in mrT3CRM not threatened patients and mrT4 patients, respectively. This was significant at *p* = 0.049.^14^ Only one study evaluated the impact of mrLNM on LR and DM.^14^ Comparators here were mrN2 versus mrN0 (LR: OR 1.158; DM: OR 1.194; *p* = 0.110) and mrN1 versus mrN0 (LR: OR 0.67; DM: OR 1.134; *p* = 0.630). No significant differences were identified.

## DISCUSSION

This meta-analysis is the first to show that mrTDs are independently associated with worse OS and DFS. In addition, mrEMVI and mrCRM involvement are independently associated with a worse OS and DFS, but mrLNMs are not. The implications of these findings are twofold. First, our findings support the routine documentation of mrTDs into standardized rectal cancer reporting templates, along with mrEMVI and mrCRM status as important prognostic markers. Second, it suggests that extrapolating the prognostic power of pathological T and N staging parameters onto MRI reporting does not accurately identify patients at high risk of recurrence or death.

Our study does have some limitations. Despite having a consistent definition for mrTDs in 11 studies included in the meta-analysis and a high-quality study assessment as per the NOS, heterogeneity was significant in the pooled assessment for mrTDs (DFS and OS) and mrEMVI (OS only). This could be due to variations in follow up, clinical diversity (especially T stage of the cohorts), and the biases associated with retrospective studies. All studies included in our meta-analysis were retrospective cohort studies. This inherently introduces selection bias and confounding variables that may not have been accounted for. Variable interobserver variability between radiologists in mrTD identification when it was evaluated, as well as incomplete details on imaging protocols utilized, could impact reliability and generalizability. This could also explain the discrepancy in mrTD prevalence seen in this meta-analysis. We have previously hypothesized a prevalence on MRI at around 26% due to its three-dimensional nature, but have observed a lower prevalence at only 19.9%. Finally, although we show that mrT stage is not independently associated with a worse DFS and OS, this was based on only three studies comparing mrT3-T4 disease with mrT2 disease. T3 disease is a heterogenous group within itself and is subclassified into a–d groups depending on distance of tumor extension into the muscularis propria (<1 mm to >15 mm). T3a and T3b disease has been shown to have a significantly better prognosis than T3c or T3d.^24^ T3c and T3d are also associated with a higher risk of EMVI and CRM positivity.^25,26^ We could not capture these important nuances in this study and therefore are hesitant to make a definitive stance with regard to mrT as a poor preoperative prognostic factor. Even so, we do advocate that mrT routinely be reported due to the increasing use of local excision and watch-and-wait treatment strategies being employed in early rectal cancer.^27^

Neoadjuvant treatment is not without risk, including long-term bowel, bladder, and sexual dysfunction quoted at 14%,^28^ and therefore should be reserved for individuals at increased risk of recurrence and death. This meta-analysis suggests that these are mrTD-, mrEMVI-, and mrCRM-positive patients, but not those who are mrLNM-positive. As a result, MRI reporting based on TNM, which prioritizes nodal staging as a whole, needs reconsideration. Several alternatives such as TDV^21^ or TE^20^ have been proposed. The TD in TDV and T in TE represent mrTDs. The V in TDV and E in TE represent mrEMVI. TDV places equal weight on mrTDs and mrEMVI and mrCRM positivity, and patients are considered high risk of recurrence if any of these are positive. Alternatively, low-risk patients are negative for all. Retrospective assessment of the performance of TDV in a cohort of patients proceeding straight to surgery showed that TDV was more capable of predicting prognosis than TNM.^21^ The Mercury 3 trial, which is due to start recruiting imminently, is primarily aimed at validating TDV, the outcomes of which are eagerly anticipated.^29^ In comparison, TE stratifies risk into levels in an attempt to assign appropriate weighting to mrTD and mrEMVI relative to each other due to their high association. A score of 0 is negative for both, 1 is mrEMVI-positive but mrTD-negative, and 2 is mrTD-positive. A TE score of 2 was better able to predict OS and DFS compared with mrLNM, mrT, and mrCRM.^20^ Unfortunately, a direct comparison with TNM was not performed.

mrEMVI was also shown to be independently associated with worse OS and DFS in our meta-analysis. The exact relationship between TDs and EMVI is unclear; however, its high association on retrospective data has led us to the theory that TDs are part of a hematogenous metastatic pathway, potentially as a form of progression of EMVI.^10,15,22^ As a result, some have considered TDs and EMVI as a single entity with regard to prognosis. We would argue against this approach, as this meta-analysis suggests that mrTDs have a higher risk of death and recurrence than mrEMVI alone. Furthermore, in studies analyzing regression of high-risk factors on MRI post neoadjuvant treatment, the presence of mrTDs at baseline is associated with decreased regression of mrEMVI.^17^ The persistence of mrTD post treatment (ymrTD) is also associated with a worse prognosis compared with EMVI persistence (ymrEMVI).^10,15^

Despite these arguments, there is a significant barrier to change, and fear from clinicians to discard previously considered gospels of management without the support of randomized controlled trials. This was highlighted with the release of the updated National Institute of Health and Care Excellence (NICE) colorectal cancer guidelines in 2020, which advocated for the use of neoadjuvant chemoradiotherapy in all node-positive patients.^30^ These guidelines are based on historic research performed prior to the use of MRI and TME surgery^28,31,32^ and were received with significant critique and backlash from clinicians, resulting in withdrawal from NICE.^33^ The American Society of Clinical Oncology (ASCO) and the European Society for Medical Oncology (ESMO) have recently updated their guidelines on locally advanced rectal cancer.^34,35^ These are more acceptable. Both recognize mrEMVI and mrCRM as poor prognostic factors that should be routinely reported. ASCO also recognizes mrTDs as a poor prognostic factor that should be included in standardized reporting, as well as a criteria for consideration for total neoadjuvant therapy. Although mrLNM remains an indication for neoadjuvant treatment, it is promising to see a shift towards the inclusion of prognostically significant TDs, EMVI, and CRM.^35^ In comparison, the ESMO acknowledges that mrTDs may also be a poor prognostic factor but states that further validation studies are required.^34^ The increasing recognition of the importance of mrTDs through published work such as this article, the actively recruiting COMET trial, which is a multicenter trial aiming to validate MRI as a tool to detect TDs, as well as confirming prognostic outcomes, should aid in breaking down these barriers.^36^ Patients with mrTD and mrEMVI must be selectively stratified for neoadjuvant treatments in future clinical trials to further consolidate this shift.

## Conclusion

This systematic review and meta-analysis provides the first quantitative evidence that mrTDs in rectal cancer are independently associated with significantly worse OS and DFS. It also identifies mrEMVI and mrCRM status as poor preoperative prognostic factors, but mrLNM status did not affect prognosis. These findings advocate for the routine reporting of these poor prognostic features on MRI in rectal cancer patients and challenges the current TNM staging system, which stratifies patient risk on the basis of mrT and mrLNM findings that have no independent prognostic power. A more accurate method of staging is needed to fully harness the ability of MRI to categorize patients into prognostic groups and therefore guide treatment in a meaningful way.

## Supplementary Information

Below is the link to the electronic supplementary material.

Supplementary file 1 (DOCX 190 KB)

## Data Availability

The datasets used and/or analyzed during the current study are available from the corresponding author on reasonable request.
