# Supplementary material for: The Prognostic Impact of Imaging Detected Tumor Deposits in Rectal Cancer: A Systematic Review and Meta-analysis
Source: Ann Surg Oncol. 2025 Sep 30;33(1):199–209. doi: 10.1245/s10434-025-18371-w (PMC12689810; doi:10.1245/s10434-025-18371-w)
Supplement: Supplementary file 1 — (DOCX 190 KB) [file 10434_2025_18371_MOESM1_ESM.docx]

| **Database** | **Time span** | **Search strategy** |
| --- | --- | --- |
| MEDLINE | 1946 to 10^th^ March 2025 | 1 Tum?r deposit*.mp.  2 (extranodal adj2 deposit*).mp.  3 (extramural adj2 deposit*).mp.  4 1 or 2 or 3  5 exp Extranodal Extension  6 4 or 5  7 Magnetic Resonance Imaging  8 MRI.mp.  9 mrTD.mp.  10 7 or 8 or 9  11 overall survival.mp.  12 disease free survival.mp.  13 local recurr*nce.mp.  14 distant metastas*s.mp.  15 11 or 12 or 13 or 14  16 exp Colorectal Neoplasms/  17 colorectal cancer.mp.  18 bowel cancer.mp.  19 rectal cancer.mp.  20 16 or 17 or 18 or 19  21 6 and 10 and 15 and 20 |
| EMBASE (OvidSP) | 1947 to 10^th^ March 2025 | 1  Tumo?r deposit*.mp.  2  (extranodal adj2 deposit*).mp  3  (extramural adj2 deposit*).mp.  4  1 or 2 or 3 5  MRI.mp.  6  mrTD.mp.  7  magnetic resonance imaging.mp.  8  6 or 7 or 8  9  Overall survival.mp.  10  disease free survival.mp.  11  local recurr*nce.mp.  12  distant metastas*s.mp.  13  9 or 10 or 11 or 12  14  rectum tumor/ or rectum carcinoma/ or colorectal cancer/ or colon carcinoma/ or rectum cancer/  15  bowel cancer.mp.  16  14 or 15  17  4 and 8 and 13 and 16 |
| Scopus | January 1950 to April 2020 | ( rectal AND cancer ) OR ( bowel AND cancer ) OR ( colorectal AND cancer ) AND ( distant AND metastas*s ) OR ( local AND recurr*nce ) OR ( disease AND free AND survival ) ( overall AND survival ) AND ( magnetic AND resonance AND imaging ) OR ( MRI ) OR ( mrTD ) AND ( extramural W/2 deposit* ) OR ( extranodal W/2 deposit* ) OR ( tumour AND deposit ) |

**Table S1:** Detailed Search Strategy


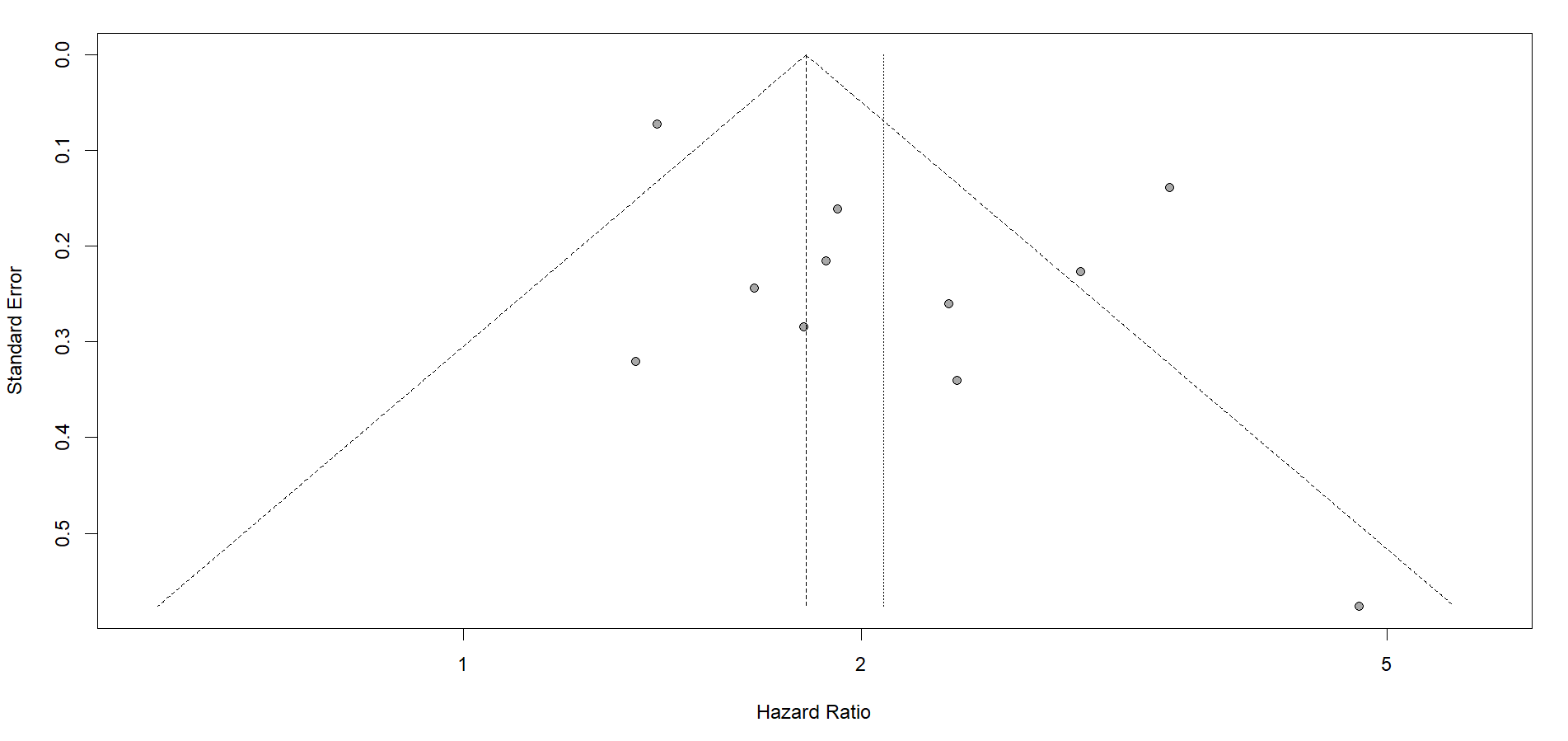


**Figure S1:** Funnel plot assessing publication bias for the 11 included studies in the meta-analysis. Eggers test: t = 1.70, df = 9, p-value = 0.1234


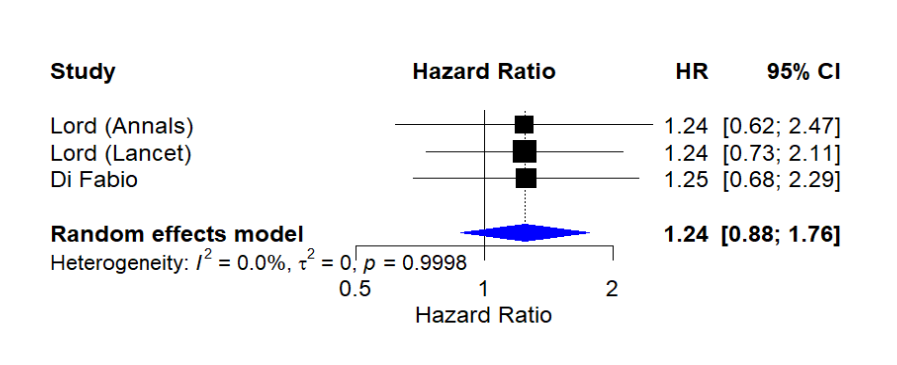


**Figure S2:** Forest plot showing pooled Hazard ratio (HR) for MRI detected T3-4 vs T2. Prognostic measure is Overall Survival (OS). P=0.21.


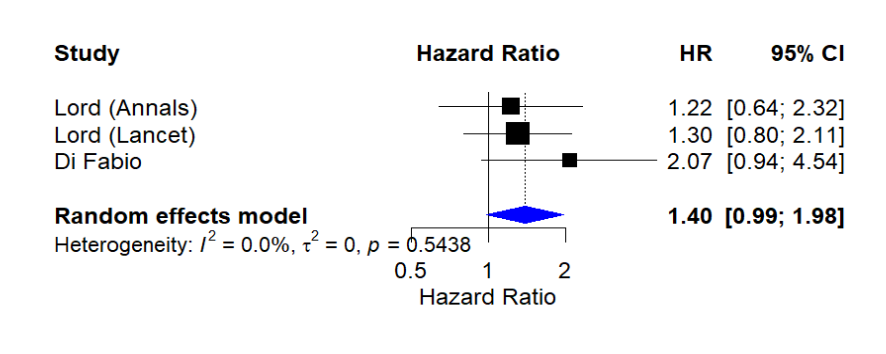


**Figure S3:** Forest plot showing pooled Hazard ratio (HR) for MRI detected T3-4 vs T2. Prognostic measure is Disease Free Survival (DFS). P=0.21.
